# Supplementary figures and images for: The aetiology and clinical characteristics of cryptococcal infections in Far North Queensland, tropical Australia
Source: PLoS One. 2022 Mar 30;17(3):e0265739. doi: 10.1371/journal.pone.0265739 (PMC8966997; doi:10.1371/journal.pone.0265739)

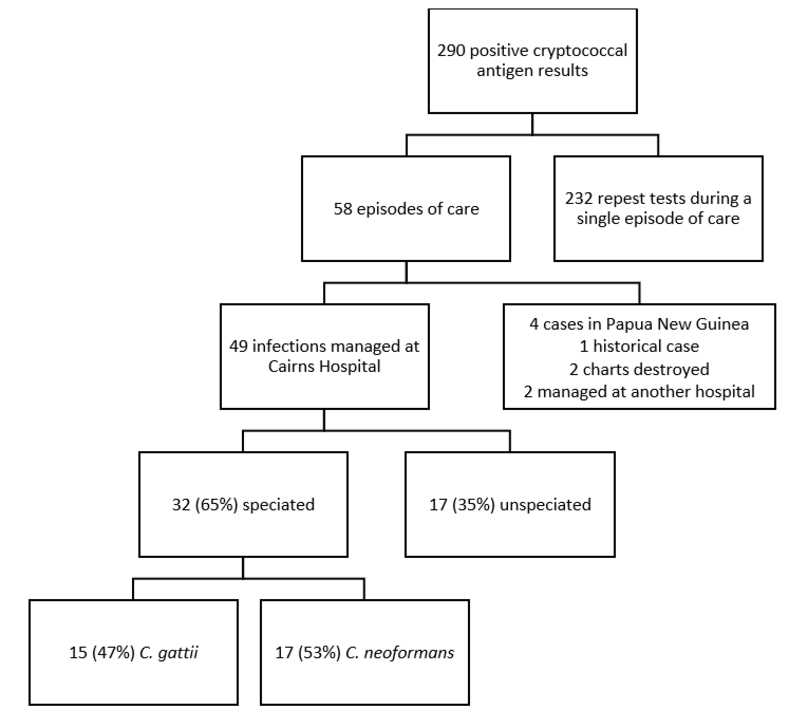

Supplement: S1 Fig — (TIF) [file pone.0265739.s002.tif]

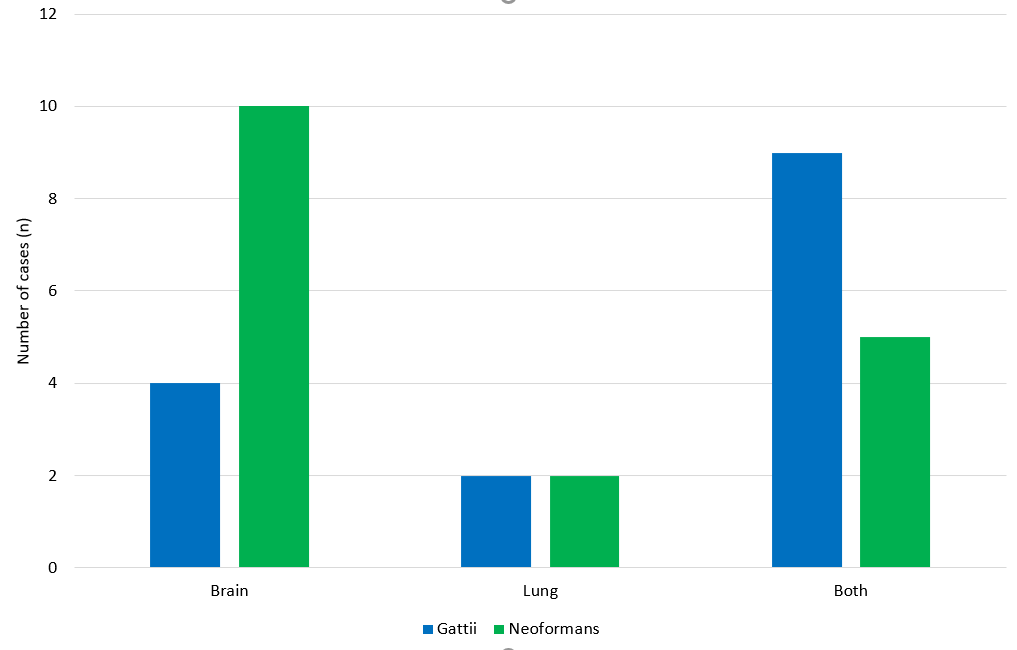

Supplement: S2 Fig — gattii and C. neoformans infection. (TIF) [file pone.0265739.s003.tif]
