# Supplementary material for: The aetiology and clinical characteristics of cryptococcal infections in Far North Queensland, tropical Australia
Source: PLoS One. 2022 Mar 30;17(3):e0265739. doi: 10.1371/journal.pone.0265739 (PMC8966997; doi:10.1371/journal.pone.0265739)
Supplement: S1 Table — (DOCX) [file pone.0265739.s004.docx]

**S1 Table. Imaging findings in patients with pulmonary disease**

|  | ***C. gattii* with lung involvement ^a^ n = 11** | ***C. neoformans* with lung involvement ^a^ n = 6** | **p** |
| --- | --- | --- | --- |
| **Had chest x-ray** | 11 (100%) | 6 (100%) |  |
| **Normal** | 2 (18%) | 0 | 0.52 |
| **Cryptococcoma ^b^** | 7 (64%) | 3 (50%) | 0.64 |
| **Size of lesion** | 57 (36-73) | 60 (46-74) | 0.51 |
| **Pleural effusion** | 0 | 0 | - |
| **Lymphadenopathy** | 0 | 1 (17%) | 0.40 |
| **Had CT chest** | 9 (82%) | 5 (83%) |  |
| **Normal** | 0 | 0 |  |
| **Cryptococcoma ^b^** | 8 (89%) | 3 (60%) | 0.51 |
| **Size of lesion** | 47 (39-75) | 44 (12-75) | 0.66 |
| **Multiple lesions** | 2 (22%) | 2 (40%) | 0.24 |
| **Cavitating lesions** | 0 | 0 | - |
| **Pleural effusion** | 0 | 0 | - |
| **Lymphadenopathy** | 1 (11%) | 1 (20%) | 1.0 |

**^a^** Although 33 patients in the cohort had lung involvement, in only 17 was speciation possible
^b^ Cryptococcomas defined as lesions > 10mm.
