# Supplementary material for: The aetiology and clinical characteristics of cryptococcal infections in Far North Queensland, tropical Australia
Source: PLoS One. 2022 Mar 30;17(3):e0265739. doi: 10.1371/journal.pone.0265739 (PMC8966997; doi:10.1371/journal.pone.0265739)
Supplement: S2 Table — (DOCX) [file pone.0265739.s005.docx]

**S2 Table. Imaging findings in patients with CNS disease**

|  | ***C. gattii* with CNS involvement n = 13** | ***C. neoformans* with CNS involvement n = 15** | **p** |
| --- | --- | --- | --- |
| **Had CT brain** | 7 (54%) | 9 (60%) |  |
| **Normal** | 4 (57%) | 4 (44%) | 1.0 |
| **Cryptococcoma ^c^** | 2 (29%) | 0 | 0.18 |
| **Size of lesion (mm)** ^b^ | 30 | 8 | - |
| **Multiple lesions** | 0 | 1 | 0.33 |
| **Hydrocephalus** | 0 | 0 | 1.0 |
| **Had MRI brain** | 8 (62%) | 5 (33%) |  |
| **Normal** | 1 (13%) | 2 (40%) | 0.51 |
| **Cryptococcoma ^c^** | 3 (38%) | 0 | 0.23 |
| **Size of lesion (mm)** ^b^ | 10 | 8 | - |
| **Multiple lesions** | 2 (25%) | 0 | 0.49 |
| **Hydrocephalus** | 0 | 0 | 1.0 |

^a^ Although 33 patients in the cohort had brain involvement, in only 28 was speciation possible

^b^ Median value presented; the small number of cases in which the precise size of the lesion was reported and in which the films were unavailable for examination, precluded reliable determination of the interquartile range or statistical comparison of the species.

^c^ Cryptococcomas defined as lesions >10mm.
