# Supplementary material for: The aetiology and clinical characteristics of cryptococcal infections in Far North Queensland, tropical Australia
Source: PLoS One. 2022 Mar 30;17(3):e0265739. doi: 10.1371/journal.pone.0265739 (PMC8966997; doi:10.1371/journal.pone.0265739)
Supplement: S3 Table — (DOCX) [file pone.0265739.s006.docx]

**S3 Table. Symptoms of the patients and their vital signs at presentation, stratified by Cryptococcal species.**

|  | ***C. gattii* n=15** | ***C. neoformans* n=17** | **P** |
| --- | --- | --- | --- |
| **Dyspnoea ^a^** | 3/11 (27%) | 2/6 (33%) | 1.0 |
| **Cough ^a^** | 6/11 (55%) | 3/6 (50%) | 1.0 |
| **Pleurisy ^a^** | 3/11 (27%) | 1/6 (17%) | 1.0 |
| **Headache ^b^** | 11/13 (85%) | 13/15 (87%) | 1.0 |
| **Photophobia ^b^** | 3/13 (23%) | 3/15 (20%) | 1.0 |
| **Meningism ^b^** | 6/13 (46%) | 7/15 (47%) | 1.0 |
| **Glasgow Coma Scale score ^b^** | 15 (14-15) | 15 (10-15) | 0.20 |
| **Focal neurology ^b^** | 8/13 (62%) | 7/15 (47%) | 0.48 |
| **Seizure ^b^** | 2/13 (15%) | 2/15 (13%) | 1.0 |
| **Myalgias ^c^** | 1/15 (7%) | 6/17 (35%) | 0.09 |
| **Sweats and fevers ^c^** | 9/15 (60%) | 7/17 (41%) | 0.48 |
| **Weight loss / Nausea / Vomiting ^c^** | 10/15 (67%) | 10/17 (59%) | 0.73 |
| **Heart rate (beats/minute) ^c^** | 73 (61-88) | 82 (75-90) | 0.15 |
| **Systolic blood pressure (mmHg) ^c^** | 133 (118-162) | 130 (110-155) | 0.41 |
| **Oxygen saturations on room air (%) ^c^** | 98 (97-100) | 99 (98-100) | 0.73 |
| **Respiratory rate (breaths/minute) ^c^** | 18 (18-20) | 16 (16-20) | 0.58 |
| **Temperature (◦C) ^c^** | 37.0 (36.7-38.0) | 36.7 (36.5-37.0) | 0.06 |

^a^ Among the 17 patients with lung involvement in whom the cryptococcus could be speciated
^b^ Among the 28 patients with CNS involvement in whom the cryptococcus could be speciated
^c^ Symptoms and signs at presentation among all 32 patients in whom speciation was performed.

Data presented as absolute number (%) or median (IQR).

Denominator is determined by site of infection i.e. *C. gattii* patients: 13 with cerebral involvement, 11 with pulmonary involvement; *C. neoformans*: 15 with cerebral involvement, 6 with pulmonary involvement.
