# Supplementary material for: The aetiology and clinical characteristics of cryptococcal infections in Far North Queensland, tropical Australia
Source: PLoS One. 2022 Mar 30;17(3):e0265739. doi: 10.1371/journal.pone.0265739 (PMC8966997; doi:10.1371/journal.pone.0265739)
Supplement: S4 Table — (DOCX) [file pone.0265739.s007.docx]

**S4 Table. Laboratory findings of the patients at presentation stratified by Cryptococcal species.**

|  | ***C. gattii***  **n=15** | ***C. neoformans***  **n=17** | **p** |
| --- | --- | --- | --- |
| **Haemoglobin (x10^9^/L)** | 132 (123-150) | 121 (111-146) | 0.12 |
| **White cell count (x10^9^/L)** | 10.1 (8.4-14.7) | 9.2 (6.4-14.9) | 0.16 |
| **Platelets (x10^9^/L)** | 277 (221-346) | 237 (191-343) | 0.24 |
| **Neutrophils (x10^9^/L)** | 8.3 (6.2-13.1) | 7.0 (5.2-12.4) | 0.36 |
| **Lymphocytes (x10^9^/L)** | 1.4 (0.9-2.3) | 0.8 (0.5-1.3) | 0.04 |
| **C-reactive protein** | 15 (9-36) | 21 (5-40) | 0.87 |
| **Sodium (mmol/L)** | 134 (127-137) | 134 (128-136) | 0.82 |
| **Potassium (mmol/L)** | 3.8 (3.3-3.9) | 3.9 (3.6-4.2) | 0.56 |
| **Chloride (mmol/L)** | 96 (92-101) | 98 (88-101) | 0.98 |
| **Bicarbonate (mmol/L)** | 26 (24-28) | 26 (25-29) | 0.36 |
| **Glucose (mmol/L)** | 7.6 (5.9-8.8) | 6.1 (5.4-11.2) | 0.60 |
| **Urea (mmol/L)** | 5.1 (3.2-7.6) | 4.2 (2.7-6.8) | 0.78 |
| **Creatinine (µmol/L)** | 83 (68-100) | 64 (50-96) | 0.12 |
| **Albumin g/dL** | 38 (35-43) | 35 (31-41) | 0.37 |
| **Protein g/dL** | 77 (70-85) | 73 (65-81) | 0.34 |
| **Globulin g/dL** | 38 (31-45) | 36 (26-41) | 0.33 |
| **Bilirubin (µmol/L)** | 10 (8-15) | 10 (6-15) | 0.45 |
| **ALP (IU/L)** | 81 (62-91) | 70 (55-91) | 0.84 |
| **GGT (IU/L)** | 27 (18-48) | 32 (18-53) | 0.50 |
| **ALT (IU/L)** | 22 (10-34) | 18 (14-27) | 0.71 |
| **AST (IU/L)** | 27 (14-42) | 21 (17-31) | 0.60 |
| **LDH (IU/L)** | 225 (173-331) | 223 (172-268) | 0.73 |

Data presented as median (IQR)
