# Supplementary material for: The aetiology and clinical characteristics of cryptococcal infections in Far North Queensland, tropical Australia
Source: PLoS One. 2022 Mar 30;17(3):e0265739. doi: 10.1371/journal.pone.0265739 (PMC8966997; doi:10.1371/journal.pone.0265739)
Supplement: S5 Table — (DOCX) [file pone.0265739.s008.docx]

**S5 Table. Lumbar puncture findings stratified by Cryptococcal species.**

|  | ***C. gattii* n=12** | ***C. neoformans* n=15** | **p** |
| --- | --- | --- | --- |
| **CSF white cell count (x10^9^/L)** | 110 (29-165) | 70 (45-117) | 0.58 |
| **CSF polymorphonucleocytes (%)** | 30 (11-50) | 10 (6-49) | 0.55 |
| **CSF protein** | 920 (590-1400) | 970 (490-1900) | 0.64 |
| **CSF glucose (mmol/L)** | 2.0 (0.3-3.4) | 1.6 (0.6-3.4) | 0.98 |
| **LP opening pressure (cm H_2_O)** | 27 (16-34) | 32 (16-35) | 0.70 |
| **India ink positive** | 8/10 (80%) | 11/16 (69%) | 0.67 |
| **Number of lumbar punctures to culture negativity** | 2 (1-3) | 2 (2-3) | 0.51 |
| **Number of lumbar punctures to normal opening pressure** | 7 (0-11) | 2 (0-14) | 0.83 |

Data presented as absolute number (%) or median (IQR).

Lumbar punctures (LP) were performed in 12 patients with *C. gattii* and 15 patients with *C. neoformans*.

CSF: cerebrospinal fluid.
