# Supplementary material for: The aetiology and clinical characteristics of cryptococcal infections in Far North Queensland, tropical Australia
Source: PLoS One. 2022 Mar 30;17(3):e0265739. doi: 10.1371/journal.pone.0265739 (PMC8966997; doi:10.1371/journal.pone.0265739)
Supplement: S6 Table — (DOCX) [file pone.0265739.s009.docx]

**S6 Table. Antifungal doses and duration stratified by Cryptococcal species.**

|  | ***C. gattii***  n=13 | ***C. neoformans*** n=15 | ***P*** |
| --- | --- | --- | --- |
| **Induction with liposomal amphotericin** | 9 (69%) | 10 (67%) | 1.0 |
| **Daily dose liposomal amphotericin (mg/kg)** | 3.5 (3.3-3.8) | 3.6 (3.2-3.6) | 0.79 |
| **Induction with conventional amphotericin** | 4 (31%) | 5 (33%) | 1.0 |
| **Daily dose conventional amphotericin (mg/kg)** | 0.7 (0.6-1.0) | 0.7 (0.6-0.8) | 1.0 |
| **Induction duration (weeks)** | 5 (2-6) | 3 (2-6) | 0.25 |
| **Daily dose fluconazole (mg)** | 400 (400-800) | 400 (400-800) | 0.43 |
| **Consolidation duration (months)** | 12 (4-lifelong) | 10 (6-17) | 0.80 |

Data presented as absolute number (%) or median (IQR)
