# Supplementary material for: The aetiology and clinical characteristics of cryptococcal infections in Far North Queensland, tropical Australia
Source: PLoS One. 2022 Mar 30;17(3):e0265739. doi: 10.1371/journal.pone.0265739 (PMC8966997; doi:10.1371/journal.pone.0265739)
Supplement: S7 Table — (DOCX) [file pone.0265739.s010.docx]

**S7 Table: Characteristics and clinical course of the patients with Cryptococcus gattii infection.**

| **Case** | **Year** | **Age, gender** | **Indigenous Australian** | **Immunocompromised** | **CCI** | **Site** | **Presentation** | **Treatment** | **Outcome** |
| --- | --- | --- | --- | --- | --- | --- | --- | --- | --- |
| **1** | 1999 | 18 F | No | No | 4 | CNS | Headache, meningism, ALOC, focal neurology, fevers, weight loss | Induction AmB & 5FC for 8 weeks, consolidation fluconazole indefinitely | Survived |
| **2** | 2002 | 64 M | Yes | No | 2 | Both | Headache, focal neurology, weight loss | Induction AmB for 2 weeks, consolidation fluconazole for 3 months | Survived |
| **3** | 2003 | 49M | No | No | 0 | CNS | Headache, meningism, seizures, fevers, weight loss | Induction LAmB & 5FC for 2 weeks, consolidation fluconazole for 12 months | Survived |
| **4** | 2003 | 82 M | Yes | No | 4 | Both | Impaired consciousness, focal neurology | Induction LAmB & 5FC for 3 weeks, consolidation fluconazole indefinitely | Survived |
| **5** | 2005 | 38 M | No | No | 0 | CNS | Headache, meningism, focal neurology, new visual impairment, weight loss | Induction AmB & 5FC for 4 weeks, consolidation fluconazole | Survived |
| **6** | 2008 | 51 M | Yes | No | 2 | Both | Headache, meningism, Impaired consciousness, focal neurology, new visual impairment, weight loss | Induction AmB & 5FC for 8 weeks, consolidation fluconazole | Died |
| **7** | 2010 | 29 F | Yes | No | 0 | Both | Dyspnoea, cough, headache, meningism, fevers, weight loss | Induction LAmB & 5FC for 6 weeks, consolidation fluconazole for 12 months | Survived |
| **8** | 2010 | 49 F | Yes | No | 0 | Both | Cough, headache | Induction LAmB & 5FC for 5 weeks | Survived |
| **9** | 2014 | 63 M | No | No | 0 | CNS | Headache, focal neurology, fevers, weight loss | Induction LAmB & 5FC for 2 weeks, consolidation fluconazole for 6 months | Survived |
| **10** | 2016 | 32 F | Yes | No | 0 | Both | Pleurisy, headache, meningism, focal neurology, new visual impairment, papilloedema, myalgias, fevers, weight loss | Induction LAmB & 5FC for 3 weeks, consolidation fluconazole for 8 months | Survived |
| **11** | 2018 | 51 F | Yes | No | 8 | Lung | Cough | Oral fluconazole for 12 months | Survived |
| **12** | 2019 | 19 M | Yes | No | 0 | Both | Cough, pleurisy, headache, hearing loss, weight loss | Induction LAmB & 5FC for 6 weeks | Survived |
| **13** | 2019 | 53 F | Yes | No | 2 | Both | Pleurisy, headache, meningism, Impaired consciousness, focal neurology, hearing loss, fevers, weight loss | Induction LAmB & 5FC for 6 weeks, consolidation fluconazole for 18 months | Survived |
| **14** | 2019 | 60 M | Yes | No | 5 | Lung | Dyspnoea, cough, headache, fevers | Oral fluconazole for 2 months | Survived |
| **15** | 2019 | 29 F | No | No | 0 | Both | Dyspnoea, cough, seizures, fevers | Induction LAmB & 5FC for 6 weeks, consolidation fluconazole for 18 months | Survived |

Patients received 5-flucytosine (5FC) and either liposomal amphotericin B (LAmB) or conventional amphotericin B (AmB) as induction therapy and fluconazole as consolidation. CCI: Charlson Comorbidity Index. CNS: Central nervous system.
