# Supplementary material for: The aetiology and clinical characteristics of cryptococcal infections in Far North Queensland, tropical Australia
Source: PLoS One. 2022 Mar 30;17(3):e0265739. doi: 10.1371/journal.pone.0265739 (PMC8966997; doi:10.1371/journal.pone.0265739)
Supplement: Attachment — Submitted filename: Comments PLOS One 2021.docx [file pone.0265739.s011.docx]

In this manuscript, Sim et al. summarized the etiology, epidemiology and clinical course of cryptococcal infections in Far North Queensland between 1999 and 2019. During this period, infections with both *Cryptococcus gattii* and *C. neoformans* were identified. Individuals infected with *C. gattii* were less likely to be immunocompromised compared to patients with *C. neoformans*. Interestingly, they found that nearly 90% of the *C. gattii* infections had CNS involvement. Patients with *C. gattii* infections were more likely to be current or former smoker than patients with *C. neoformans*. Furthermore, more than 50% of the patients had a history of hazardous alcohol use. These clinical data are interesting; however, the manuscript will definitively benefit from an expanded mechanistic discussion on these clinical observations.

Comments

1) In this manuscript, 90% of the *C. gattii* infections had CNS involvement. Recent mouse studies showed that the primary target organ of *C. gattii* is different from that of *C. neoformans*. *C. gattii* grows faster in the lung and is primarily linked to pulmonary diseases (Pubmed PMID: 22570277). The authors should expand the discussion on this point.
2) Patients with *C. gattii* infections more likely to be smoker. It would be nice to have more discussion about that point.
3) It is also interesting that more than 50% of the patients had a history of hazardous alcohol use. The authors should discuss more on the potential mechanisms behind this observation. Actually, recent studies have shown that Liver cirrhosis is an independent risk factor of cryptococcal meningitis (Pubmed PMID: 25747471) and that liver macrophages play a prominent role to filter disseminating Cryptococcal yeast cells (Pubmed PMID: 31594939).
