# Supplementary material for: The aetiology and clinical characteristics of cryptococcal infections in Far North Queensland, tropical Australia
Source: PLoS One. 2022 Mar 30;17(3):e0265739. doi: 10.1371/journal.pone.0265739 (PMC8966997; doi:10.1371/journal.pone.0265739)
Supplement: Attachment — Submitted filename: Response to reviewers.docx [file pone.0265739.s012.docx]

**Response to reviewers**

We thank the Editorial staff and the reviewers for the time that they have taken to review our manuscript and the very helpful comments that they have made to improve the work. Please find below our point-by-point responses to their comments. For clarity, the Editorial staff and reviewers’ comments are presented in blue normal text, while our responses are in black normal text. Any modified - or highlighted - text is presented as black and italicized.

**Editorial staff comments**

1. Please ensure that your manuscript meets PLOS ONE's style requirements, including those for file naming. The PLOS ONE style templates can be found at 
<https://journals.plos.org/plosone/s/file?id=wjVg/PLOSOne_formatting_sample_main_body.pdf> and 
<https://journals.plos.org/plosone/s/file?id=ba62/PLOSOne_formatting_sample_title_authors_affiliations.pdf>

**Response:** We have checked that the manuscript meets the style requirements - including file naming - and feel that it does. We are very happy to address specific concerns if any remain.

2. Please provide additional details regarding participant consent. In the ethics statement in the Methods and online submission information, please ensure that you have specified what type you obtained (for instance, written or verbal, and if verbal, how it was documented and witnessed). If your study included minors, state whether you obtained consent from parents or guardians. If the need for consent was waived by the ethics committee, please include this information.

**Response:** Requirement for informed consent was waived by the Ethics Committee as we highlight in lines 126-128.

*“The FNQ Human Research Ethics Committee provided ethical approval for the study (HREC/16/QCH/110 – 1083 LR). As the data were retrospective and de-identified, the Committee waived the requirement for informed consent”.*

3. We note that Figures 1 and 4 in your submission contain [map/satellite] images which may be copyrighted. All PLOS content is published under the Creative Commons Attribution License (CC BY 4.0), which means that the manuscript, images, and Supporting Information files will be freely available online, and any third party is permitted to access, download, copy, distribute, and use these materials in any way, even commercially, with proper attribution. For these reasons, we cannot publish previously copyrighted maps or satellite images created using proprietary data, such as Google software (Google Maps, Street View, and Earth). For more information, see our copyright guidelines: <http://journals.plos.org/plosone/s/licenses-and-copyright>.
 
We require you to either (1) present written permission from the copyright holder to publish these figures specifically under the CC BY 4.0 license, or (2) remove the figures from your submission:
**Response:** All the maps in the submission were created using constructed using mapping software (MapInfo version 15.02, Connecticut, USA) using data provided by the State of Queensland (QSpatial) which are not copyrighted. Queensland Place Names —State of Queensland (Department of Natural Resources, Mines and Energy) 2019, available under Creative Commons Attribution 4.0 International licence <https://creativecommons.org/licenses/by/4.0/> and ‘Coastline and state border–Queensland - State of Queensland (Department of Natural Resources, Mines and Energy) 2019, available under Creative Commons Attribution 4.0 International licence <https://creativecommons.org/licenses/by/4.0/>

These data - which are freely available in the public domain - are provided by the State Government of Queensland. This is a similar situation to some of the U.S. government sources that were suggested in the decision email.

All the patients were managed in the Queensland State Government’s Public Health system. All the authors contributed to the manuscript when employed by the State Government of Queensland.

We have used these data to create maps in multiple PLoS publications in the past 4 years without any issue.

1. PLoS Negl Trop Dis. 2021 Jun 21;15(6):e0009544. doi: 10.1371/journal.pntd.0009544. eCollection 2021 Jun.

2. PLoS Negl Trop Dis. 2021 Jan 14;15(1):e0008990. doi: 10.1371/journal.pntd.0008990. eCollection 2021 Jan.

3. PLoS One. 2020 Sep 3;15(9):e0238719. doi: 10.1371/journal.pone.0238719. eCollection 2020.

4. PLoS Negl Trop Dis. 2019 Jul 18;13(7):e0007583. doi: 10.1371/journal.pntd.0007583. eCollection 2019 Jul.

5. PLoS Negl Trop Dis. 2019 Feb 13;13(2):e0007205. doi: 10.1371/journal.pntd.0007205. eCollection 2019 Feb.

6. PLoS Negl Trop Dis. 2017 Mar 6;11(3):e0005411. doi: 10.1371/journal.pntd.0005411. eCollection 2017

We have added a footnote to Figures 1 and 4 - specifically addressing this copyright issue - to make this clearer.

4. Please include a copy of Table 2 which you refer to in your text on page 10.

**Response:** We thank the Editorial staff for highlighting this typographical error in which we had misnumbered the table in the original submission. This table is table 3 in the revised manuscript (page 13).

**Reviewer comments**

Reviewer #1: This paper reports the retrospective experience with 49 patients hospitalized in Northern Queensland with cryptococcosis. The report centers around 32 cases in which the isolate was identified by species: C neoformans or C. gattii. No novel findings were encountered. The higher prevalence of immunosuppressed patients with C. neoformans than C. gattii in Australia was confirmed. Some of the other difference between the two species in other reports were not found, in part due to the small sample size, such as more infections confined to the lung or more mass lesions in the brain among C. gattii cases.
The paper is burdened with masses of data that cannot be interpreted. The speculated connection between the higher prevalence of C. gattii cases in the last half of the year and flowering in Eucalyptus trees is specious. The lower, though normal, lymphocyte counts in the peripheral blood of C. neoformans patients is an example of applying a p value of 0.05 to more than 20 comparisons and finding one value of 0.04. There are larger and more detailed analyses of MRI and CT images, antifungal susceptibility and clinical descriptions of cryptococcosis in Australia than in this report. The long and speculative Discussion in the paper needs drastic shortening. The authors might consider deleting the data on the 17 cases with unspeciated isolates and minimizing speculation. If further analyses can provide insights into how cryptococcosis might differ in indigenous Australians, that might be instructive. Cairns has an experience with that population. Just because no difference was found doesn’t mean there were no differences.

**Response**: We thank the reviewer for their constructive comments. We agree that the discussion was too long and have shortened it significantly, by almost 30% (1217 to 864 words), as suggested.

We also agree with their comments about the limitations of the study which are largely a product of its sample size. Indeed, we highlight this specifically in lines 336-337. The risk of type 2 errors that we highlight in the limitations section (line 340) states precisely what the reviewer is suggesting. “Just because no difference was found doesn’t mean there were no differences” is the definition of a type 2 error.

Certainly, while there are other Australian analyses of larger size, this is the largest ever analysis in a tropical Australian location. The only comparable study - contained only 18 patients, only 12 of which were *C. gattii* - from the Northern Territory (Jenney et al. 2004, reference 7), was published over 15 years ago and examined patients managed between 1993 and 2000. The management of critical illness and cryptococcal disease has evolved since then. The significant difference in case-fatality rate between the two series highlights this and provides updated and contemporary data for the reader.

The fact that Indigenous Australians were over-represented in our cohort is also significant, and the reviewer’s comment regarding further analysis in this population is insightful. Further analyses have been presented as suggested (lines 147-156, lines 300-305 and table 2).

The reviewer suggests that the paper is “burdened with masses of data” but it is important to note that most of this data is presented as supplementary material (7 supplementary tables, 2 supplementary figures and the dataset) which we present for the interested reader. There are only 3 tables of data in the revised submission which includes an additional table examining the burden of disease in Indigenous Australians as suggested by the reviewer.

The reviewer is correct to note that for every 20 analyses of a normal population, 1 - on average - will, by chance, have a p value of <0.05. However, we disagree with the reviewer when they state that the “lower, though normal, lymphocyte counts in the peripheral blood of C. neoformans patients is an example of applying a p value of 0.05 to more than 20 comparisons and finding one value of 0.04.” The median (IQR) lymphocyte count in patients with *C. neoformans* was 0.8 (0.5-1.3) x 10^6^/L. The median lymphocyte count is below the normal reference range for the lymphocyte count in our laboratory (1.0-4.0 x 10^6^/L), so the majority of patients with *C. neoformans* had lymphopenia. This is unlikely to be due to chance as 8/17 patients with confirmed *C. neoformans* had established immunocompromise (including 3 patients with advanced HIV - with lymphocyte counts of 0.1, 0.3 and 0.4 x 10^6^/L respectively - and 2 patients with solid organ transplants with lymphocyte counts of 0.3 and 0.7 x 10^6^/L respectively. This would accord with the knowledge that *C. neoformans* is a disease of immunodeficiency/immunocompromise and the lymphopenia is a marker of this, rather than a chance finding from multiple analyses.

We acknowledge the reviewer’s caution in suggesting correlation between observed data – it is important not to overstate our findings. Indeed, we specifically highlight this point in lines 344-345. We agree that it is important not to attribute causation between the flowering of the Eucalypt trees and cryptococcosis. Indeed, we are pains to make this point (lines 312-313).

The association between Eucalyptus trees (specifically *E. tereticornis* and *E. camaldulensis*) is very well established (Ellis DH, Pfeiffer TJ J Clin Microbiol. 1990;28(7):1642., Sorrell TC, Chen SC, Ruma P, Meyer W, Pfeiffer TJ, Ellis DH, Brownlee AG J Clin Microbiol. 1996;34(5):1253.) so we would respectfully disagree with the reviewer who suggests that the analysis is “specious”. The data are presented only as hypothesis generating and would be, we believe, to be of interest to the readers of the article who would generally be only those with an interest in cryptococcal infection. In the large rural FNQ region there is an opportunity to look at a potential association between flowering of the trees that have been shown to harbour *C. gattii*; this is our attempt to convey these data to the readers.

Reviewer #2: In this manuscript, Sim et al. summarized the etiology, epidemiology and clinical course of cryptococcal infections in Far North Queensland between 1999 and 2019. During this period, infections with both Cryptococcus gattii and C. neoformans were identified. Individuals infected with C. gattii were less likely to be immunocompromised compared to patients with C. neoformans. Interestingly, they found that nearly 90% of the C. gattii infections had CNS involvement. Patients with C. gattii infections were more likely to be current or former smoker than patients with C. neoformans. Furthermore, more than 50% of the patients had a history of hazardous alcohol use. These clinical data are interesting; however, the manuscript will definitively benefit from an expanded mechanistic discussion on these clinical observations.
Comments
1) In this manuscript, 90% of the C. gattii infections had CNS involvement. Recent mouse studies showed that the primary target organ of C. gattii is different from that of C. neoformans. C. gattii grows faster in the lung and is primarily linked to pulmonary diseases (Pubmed PMID: 22570277). The authors should expand the discussion on this point.

**Response:** We thank the reviewer for the time that they have taken to review our paper and agree that there are several points that could be expanded in the discussion. We have done our best to do this while also attempting to shorten the discussion overall as per reviewer 1.

We have expanded our discussion about lung involvement particularly its association with alcohol and tobacco use (lines 297-302). We did not add the mouse model reference as we did not feel we had the space in the discussion to do justice to this point. It is also a little beyond the scope of the paper which focuses on human, clinical cases.

2) Patients with C. gattii infections more likely to be smoker. It would be nice to have more discussion about that point.

**Response:** We thank the reviewer for highlighting this point - we have expanded the discussion accordingly (lines 297-302).

3) It is also interesting that more than 50% of the patients had a history of hazardous alcohol use. The authors should discuss more on the potential mechanisms behind this observation. Actually, recent studies have shown that Liver cirrhosis is an independent risk factor of cryptococcal meningitis (Pubmed PMID: 25747471) and that liver macrophages play a prominent role to filter disseminating Cryptococcal yeast cells (Pubmed PMID: 31594939).

**Response:** We agree that this is an interesting discussion point and we have expanded our discussion to include this (lines 297-302). We have added both of the suggested references for the interested reader.
